# Digestive system deep infiltrating endometriosis: What do we know

**DOI:** 10.1111/jcmm.17921

**Published:** 2023-08-25

**Authors:** Wenze Yin, Xiaoqing Li, Peng Liu, Yingjie Li, Jin Liu, Shan Yu, Sheng Tai

**Affiliations:** ^1^ Department of Hepatic Surgery Second Affiliated Hospital of Harbin Medical University Harbin China; ^2^ Department of Pathology Second Affiliated Hospital of Harbin Medical University Harbin China; ^3^ Laboratory of Medical Genetics Harbin Medical University Harbin China; ^4^ Department of Pathology Six Affiliated Hospital of Harbin Medical University Harbin China

**Keywords:** aetiology, digestive system, endometriosis, intestines, molecular mechanisms

## Abstract

Digestive system infiltrating endometriosis (DSIE) is an uncommon form of endometriosis in the digestive system. DSIE often occurs in the intestines (especially the sigmoid rectum), liver, gallbladder and pancreas. Clinically, DSIE presents with the same symptoms as endometriosis, including cyclic pain, bleeding and infertility, in addition to specific biliary/intestinal obstruction and gastrointestinal bleeding. Compared to general endometriosis, DSIE has unique biological behaviour and pathophysiological mechanisms. Most DSIEs are deep invasive endometrioses, characterized by metastasis to the lymph nodes and lymphatic vessels, angiogenesis, peripheral nerve recruitment, fibrosis and invasion of surrounding tissues. DSIE‐related peripheral angiogenesis is divided into three patterns: angiogenesis, vasculogenesis and inosculation. These patterns are regulated by interactions between multiple hypoxia‐hormone cytokines. The nerve growth factors regulate the extensive neurofibril recruitment in DSIE lesions, which accounts for severe symptoms of deep pain. They are also associated with fibrosis and the aggressiveness of DSIE. Cyclic changes in DSIE lesions, recurrent inflammation and oxidative stress promote repeated tissue injury and repair (ReTIAR) mechanisms in the lesions, accelerating fibril formation and cancer‐related mutations. Similar to malignant tumours, DSIE can also exhibit aggressiveness derived from collective cell migration mediated by E‐cadherin and N‐cadherin. This often makes DSIE misdiagnosed as a malignant tumour of the digestive system in clinical practice. In addition to surgery, novel treatments are urgently required to effectively eradicate this lesion.

## 1 INTRODUCTION

Endometriosis, a prevalent gynaecological condition, is characterized by the presence and growth of functional endometrial‐like tissue outside the uterus. This can lead to the endometrium leaving the uterus and invading tissues outside the uterine cavity, causing periodic pain, bleeding or infertility.^1^ The condition can manifest in various locations, including the peritoneum, chest cavity, central nervous system, peripheral nerves, digestive tract, etc.^2^ Notably, extrapelvic endometriosis often affects the gastrointestinal tract,^3^ leading to additional symptoms, such as pain, obstruction of biliary or intestinal function and gastrointestinal bleeding, depending on the location.^4^ Digestive system infiltrating endometriosis (DSIE), a type of deeply infiltrating endometriosis (DIE), exhibits more aggressive behaviour and distinct biological and pathophysiological traits compared to ordinary endometriosis. Due to the histological similarities between DSIE and malignant tumours, they are sometimes misdiagnosed in clinical practice. This article aims to summarize and discuss recent research findings on the molecular mechanisms underlying gastrointestinal endometriosis.

## 2 ANATOMICAL PATHOGENESIS OF DSIE

The most convincing etiological hypothesis regarding the cause of endometriosis is the menstrual reflux theory. Part of the menstrual blood flows backwards into the abdominal cavity through the fallopian tube. Consequently, the endometrial tissue enters the outer area of the uterine cavity through the flow of peritoneal fluid and metaplasia, invading and colonizing other tissues.^5^ Ectopic endometrial tissues can invade the liver, pancreas, gallbladder, gastrointestinal tract and other organs (Table 1). Endometriosis occurs in 21.4% of the cases, accounting for the highest percentage. The rectosigmoid junction (65.7%) is the most common site in the bowel, followed by the sigmoid colon (17.4%).^6^ Theoretically, menstrual blood and peritoneal fluid containing endometrial tissue can flow anywhere within the abdominal cavity, even reaching the thoracic cavity and localizing in other organs. However, when the patient is upright, gravity and the anatomical structure typically cause the menstrual blood and peritoneal fluid to flow towards specific areas in the abdominal cavity, such as the bottom of the bag of Douglas, the right lower quadrant at the termination of the small bowel mesentery (cecum and ileocecum junction), the superior aspect of the sigmoid mesocolon and the right paracolic gutter.^6^, ^7^ This phenomenon explains why endometriosis is more commonly found in the pelvis than in other parts of the abdominal cavity and why DSIE is predominantly located at the rectosigmoid junction.

**TABLE 1:** The location, symptoms, and treatment of DSIE.

Location	Symptom	Treatment	Authors	Ref
Ileum	Intussusception	Surgery	Jiang et al.	[171]
Gallbladder, abdominal wall	Pain	Surgery	Iafrate et al.	[172]
Hepatic cyst	Bloating	Surgery	Kouto et al.	[173]
Liver	Pain	Surgery	Bohra et al.	[174]
Liver, lung	Bronchobile leak	Surgery	Schuld et al.	[175]
Colon	Iron‐deficiency anaemia	Oestrogen‐bazedoxifene	Snyder et al.	[176]
Rectum, vagina	Bleeding, dysmenorrhea, anal pain	Surgery	Ling et al.	[177]
Rectosigmoid colon	Chronic pain	Surgery	Fragulidis et al.	[178]

Another assumption suggests that progenitor cells present in the peritoneal lining can directly differentiate into endometrium‐like cells and spread to the periphery. For example, bone marrow‐derived stem cells can directly differentiate into the ectopic endometrium and migrate to the uterus, providing a possible explanation for the appearance of DSIE.^8^

## 3 IMMUNE CELLS AND THEIR ASSOCIATED IMMUNE FACTORS IN DSIE

### 3.1 Tregs

A substantial body of evidence indicates that women with endometriosis experience alterations in the function of various immune cells. Notably, T‐cell populations are abnormally elevated in endometriotic tissues.^9^ Among these T lymphocytes, Regulatory T cells (Tregs) expressing Forkhead Box P3 (Foxp3) are particularly important, as they play vital roles in maintaining immune homeostasis.^10^ Interestingly, Tregs from women with endometriosis exhibit unique marker profiles, while their cytokine expression levels are similar to those in normal Tregs. Tregs exhibit unique characteristics at their site of origin. For example, TNFRII and CD45RO are expressed more in the abdominal fluid of individuals with DSIE, whereas CD45RA is expressed more in the peripheral blood.^11^ Tregs expressing Foxp3 inhibit the recruitment and function of other immune cells, protecting endometrial cells outside the uterus from destruction by the immune system.^12^ One study found that endometriotic lesions in the deep rectosigmoid colon express higher levels of Foxp3 compared to other types of endometriosis or in individuals without endometriosis.^13^ This increase in peritoneal Tregs in women with endometriosis may result from the attraction and activation of local chemokines and cytokines, especially CCL20 and TGF‐β.^14^ By reducing the recognition of endometriotic tissue antigens by immune cell populations, Tregs facilitate the long‐term persistence of endometriotic tissue.^15^ Therefore, exploring their tropism and activation could be a potential target for therapeutic intervention.^14^ Nonetheless, specific future therapeutic interventions based on this aspect of Tregs are yet to be investigated.

Furthermore, Tregs can secrete cytokines such as IL‐10, TGF‐β, IFN‐β, IL‐7 and IL‐15, with a significant increase in IL‐10 and TGF‐β expression observed in DSIE,^16^ especially in the intestinal region.^17^ These two cytokines likely play a role in the inhibition of effector T cells by Tregs.^18^ Interestingly, changes in Treg and NK cell‐related cytokines in rectosigmoid DIE are found to be correlated. The expression of TGFB, IL7 and IL15 shows a correlation with dyspareunia, dysmenorrhea and periodic sleep disorders, respectively.^17^ Additionally, related chemokines such as CCL17, CXCL12 and CX3CL1 have been shown to have statistically significant increases; however, their specific roles require further investigation.^19^

### 3.2 Macrophages

Macrophages in endometriosis exhibit abnormal behaviour, with increased secretion of cytokines including IL‐6, IL‐10, IL‐12 and TGF‐β1.^16^ While recent studies have shed light on the role of macrophages in endometriosis, their specific differences from other lesion locations in the digestive system have not been well described. However, in DSIE, macrophages of different phenotypes play essential roles in biological processes like fibrosis, proliferation and migration.^20^ A recent study highlighted that macrophages secrete CCL20 during co‐culture, promoting CCR6 activation in ESCs, thereby inducing ESC proliferation and migration.^21^ Moreover, the increased presence of matrix metalloproteinase‐9 (MMP‐9) co‐localized with CD68+ macrophages in the uteri of women with endometriosis indicates a higher number of macrophages associated with tissue remodelling in the normal endometrium of affected women, potentially linked to the aggressiveness of the endometrial tissue and fibrous tissue production.^22^ Meanwhile, macrophages and Tregs collaborate in secreting cytokines such as IL‐10 and TGF‐β1, regulating fibrosis in DSIE. Although no definitive studies on DSIE have been published, the current research suggests that macrophages are extremely active in the fibrosis and aggressiveness of DSIE.

A recent study discovered leucine‐rich repeat sequence‐containing G protein‐coupled receptor 5 (LGR5) cells in the endometrial tissue of patients with endometriosis. LGR5 was previously known for its presence in intestinal tissues and its significance in colon cancer liver metastasis, serving as a stem cell marker in small intestinal and hair follicle cells.^23^, ^24^, ^25^ Surprisingly, LGR5 was also detected in the endometrial tissue of women with endometriosis, especially in intestinal DIE.^26^ After conducting a biological analysis of LGR‐5 cells in endometrial tissue, Vallvé‐Juanico and Irene Cervelló et al. found that their biological markers were similar to those of the monocyte–macrophage system, indicating that these cells are not expressed as stem cells in endometriosis but rather represent a type of monocyte or its related derivatives.^23^, ^27^ Importantly, LGR‐5 in DIE exhibits a different gene expression pattern compared to other types of endometriosis.^23^ However, the specific role of LGR‐5 cells in endometriosis, especially DSIE, remains unexplored and required further investigation.

### 3.3 NK cells

NK cells can directly kill target cells. In DSIE, these cells are involved in the immune escape process of ectopic endometrial stromal cells, along with macrophages and Tregs. The activity of NK cells is diminished in the peritoneal fluid of women with DSIE, leading to immune escape during the transfer of free endometrial stromal cells into the peritoneal fluid.^16^ This reduction in NK cell activity cannot be attributed to apoptosis or decreased NK cell expression. This is because IL‐10 and TGF‐β secreted by macrophages, Tregs and platelets inhibit the activation of receptors such as natural killer group 2 member D (NKG2D), NK receptor (NKp46) and perforin. Additionally, the expression of inhibitory receptor killer cell immunoglobulin‐like receptor 2DL1 in NK cells was increased by these secreted cytokines.^28^, ^29^, ^30^ Furthermore, endometrial stromal cells secrete IL‐5, which inhibits NK cell‐killing activity.^31^ However, the inactivation mechanism of NK in DSIE remains unclear and requires further investigation.

## 4 OXIDATIVE STRESS AND DSIE

Recent studies have shown elevated iron levels and iron overload in various abdominal and pelvic fractions of patients with endometriosis, particularly in those whose disease is confined to the pelvic cavity.^32^, ^33^ Macrophages are particularly active in iron metabolism. Retrograde blood transports iron into macrophages and induces oxidative cellular damage, generating high levels of ROS and inducing cell proliferation and apoptosis.^32^ Oxidative stress and its activity in the lesions of patients with DIE.^34^ A previous study observed a correlation between the dysregulation of ROS in endometriosis and the dysregulated characteristics of ROS in tumour cells.^35^ ROS regulates oxidative stress and cell proliferation by activating the ERK pathway. Superoxide anions, hydrogen peroxide and nitric oxide (NO) play a role in cell growth in ectopic nodule biopsies.^35^ In addition, a triple‐blind clinical trial showed a significant reduction in ROS levels and decreased pain levels in women with endometriosis after vitamin C and E supplementation, which may be a new treatment modality.^36^ Notably, iron overload may affect the expression of poly ADP‐ribose polymerase 1 (PARP1), which is associated with growth inhibition and autophagy in diseased tissue cells.^37^ Also, an evidence explains the differences in oxidative stress in DSIE.^32^ Advanced oxidation protein products (AOPP) and nitrate/nitrite were only present at elevated concentrations in the peritoneal fluid of patients with DIE.^38^ Additionally, patients with intestinal involvement had higher concentrations of AOPP and nitrate/nitrite in the peritoneal fluid in the presence of intestinal involvement.^38^ This further demonstrates the higher reaction intensity of the oxygen radicals in DIE. Higher concentrations of nitrate/nitrite in the peritoneal fluid of patients with DIE reflect elevated NO levels, which may be related to the activation of endothelial NO synthase in ectopic tissues.^39^ NO alters the balance between Th1 and Th2 cells and promotes the growth of ectopic endometrial nodules.^40^ In addition, NO is associated with infertility and deep pain symptoms in patients with DIE.^41^, ^42^ Metallopeptidase domain 17 (ADAM17) was also found to be elevated in the abdominal fluid of patients with DIE and to regulate fibrosis in ectopic endometrial tissue by promoting excessive activation of Notch signalling and an increase in fibrosis markers.^43^ In addition, Mahaut Leconte showed that oxidative stress increased the ERK and mTOR/AKT pathways in mice, promoting DIE cell proliferation by analysing protein blots of them in vitro.^44^

## 5 OESTROGEN, THE RECEPTORS AND DSIE

17β‐Estradiol (E2) is a key hormone in endometriosis that regulates endometriotic tissue proliferation and inflammation by binding to the oestrogen receptor (ER). Estradiol acting in endometriotic tissue is mainly secreted locally by the endometriotic tissue.^45^ Aromatase, a cytochrome P450 responsible for aromatizing androgens to estrogens,^45^ is upregulated in endometriotic tissue.^46^ The steroidogenic acute regulatory protein (StAR), which helps cytoplasmic cholesterol move into the mitochondria, is similarly increased in endometriotic tissues and may be associated with prostaglandin E2.^45^, ^47^ 17β‐hydroxysteroid dehydrogenase 2 is associated with E2^45^ inactivation. A previous study confirmed that its expression was reduced in DIE.^48^ Both the oestrogen receptor (ER) and progesterone receptor (PR) are abundant in the smooth muscle components of DIE. Generally, the PR concentration was higher than the ER concentration. Interestingly, in colonic endometriosis, ER expression was lost.^49^ ER are split into two subsets, ERα and ERβ, both of which play an important role in the development of endometriosis.^50^ The expression of ERα is significantly higher than that of ERα in DIE, which may promote peripheral nerve recruitment and aggravate severe dysmenorrhea and deep pain symptoms in DIE patients.^51^, ^52^, ^53^ Additionally, Erα, which is usually highly expressed in DIE, can attach to oestrogen response elements in the JAG1 and Notch1 promoters. When activated by oestrogen, activates the Notch pathway in tandem, regulating lesion fibrosis and stromal cell invasiveness.^54^

## 6 BIOLOGICAL BEHAVIOUR OF DSIE AND ITS MOLECULAR MECHANISMS

### 6.1 Involvement of lymph nodes and lymphangiogenesis

Endometriotic cells were found in the pelvic lymph nodes of patients with endometriosis of the rectosigmoid colon, and lymphovascular invasion was also observed.^55^, ^56^ Recently, Koyama identified a case of ileal endometriosis with peripheral lymph node involvement.^57^ However, a controversial result was also reported in which the lymph nodes were not involved in rats with induced intestinal endometriosis. The possible reasons for this have not yet been explained.^58^ It still had a propensity for lymph node invasion or metastasis. Additionally, intestinal endometriosis‐associated lymph nodes showed significant immune activation characteristics and significantly fewer CD10+ endometrium‐associated cells in intestinal endometriosis‐associated lymph nodes than in other types of endometriosis with pelvic lymph nodes.^59^ Moreover, endometrioid cells found in the pelvic lymph nodes of patients with DIE do not express cytokeratin, suggesting that these cells may not be epithelial, but stromal in origin.^60^ These results support the hypothesis that lymphatic dissemination occurs during endometriosis. lymph node involvement may be associated with chemokine levels. Notably, two chemokine receptors, CCR7 and CXCR4, were significantly expressed in the pelvic outpost lymph nodes of rectovaginal DSIE lesions. However, no statistically significant difference was found in the number of their ligands, CXCL12, CCL19 and CCL21, suggesting that CCR7 may be associated with the risk of lymph node involvement.^61^ Additionally, Borrelli et al.^62^ found partial deletion of the BAF250a protein expressed by ARIDIA in the pelvic anterior lymph nodes of patients with rectovaginal and intestinal DSIE. In this study, 30%–40% of patients had a partial deletion of the BAF250a protein, which may represent a higher risk of malignant transformation in patients with DSIE. However, the deletion of BAF250a protein expression alone was not sufficient to induce malignancy (Table 2) and required additional concurrent genetic alterations, such as PIK3CA mutations or PTEN deletion.^63^

**TABLE 2:** Studies with lymph node metastasis around DSIE.

Location	Lymph node	Endometrioid cells	Lymphatic invasion	Multiple parts	Authors	Ref
Rectovagina	Pelvic sentinel lymph nodes	+		+	Mechsner et al.	[55]
Rectosigmoid colon	Proctosigmoid lymph nodes	+	+	−	Noël et al.	[56]
Ileum, appendix	Parileal lymph nodes	+	+	+	Koyama et al.	[57]
Bowel	Perienteric lymph nodes	+	+	−	Insabato et al.	[179]
Rectum	Perirectal lymph nodes	+	+	−	Abrao et al.	[180]
Rectum	Perirectal lymph nodes	+		**−**	Namkung et al.	[181]

Existing evidence indicate that the density of LYVE‐1 and Prox‐1 positive lymphatic vessels and the lymphatic vessel growth factor in endometriosis tissues of patients with endometriosis, especially intestinal DSIE, are significantly higher than those in normal human intestinal wall tissues.^64^, ^65^ Significantly increased expression of vascular endothelial growth factor (VEGF‐C/VEGF‐D) was observed in the epithelial and stromal cells of the intestinal endothelial ectopic tissue.^64^ These are the two main lymphangiogenic initiators that play important roles in the generation and development of lymphatic vessels.^60^ Other lymphangiogenic factors were also found in DIE lesions, such as VEGF‐A, VEGFR‐2.^66^, ^67^ Among these, VEGF‐C is the main stimulatory factor that activates VEGFR‐3 to promote lymphangiogenesis.^68^ These studies demonstrate a tendency for lymphangiogenesis in DSIE. Notably, macrophages are significantly active in endometriotic tissue, leading to elevated levels of TNF‐α and IL1‐β, which promote local upregulation of lymphatic vascular growth factor, leading to increased lymphangiogenesis and immune cell recruitment.^60^, ^69^ The phenomenon of lymphangiogenesis in intestinal endometriosis may be a way for the body to clear lesions. However, the recruitment of immune cells and lymphangiogenesis may be related to the infiltration of the lesion into the periphery through the lymphatics and the metastasis of the lesion to the lymph nodes. However, although DSIE can metastasize along lymph nodes with lymphatic vessels similar in character to malignant tumours, endometrioid cells in metastatic lymph nodes do not exhibit nuclear anisotropy.^70^, ^71^

### 6.2 Vascularization

**FIGURE 1:**
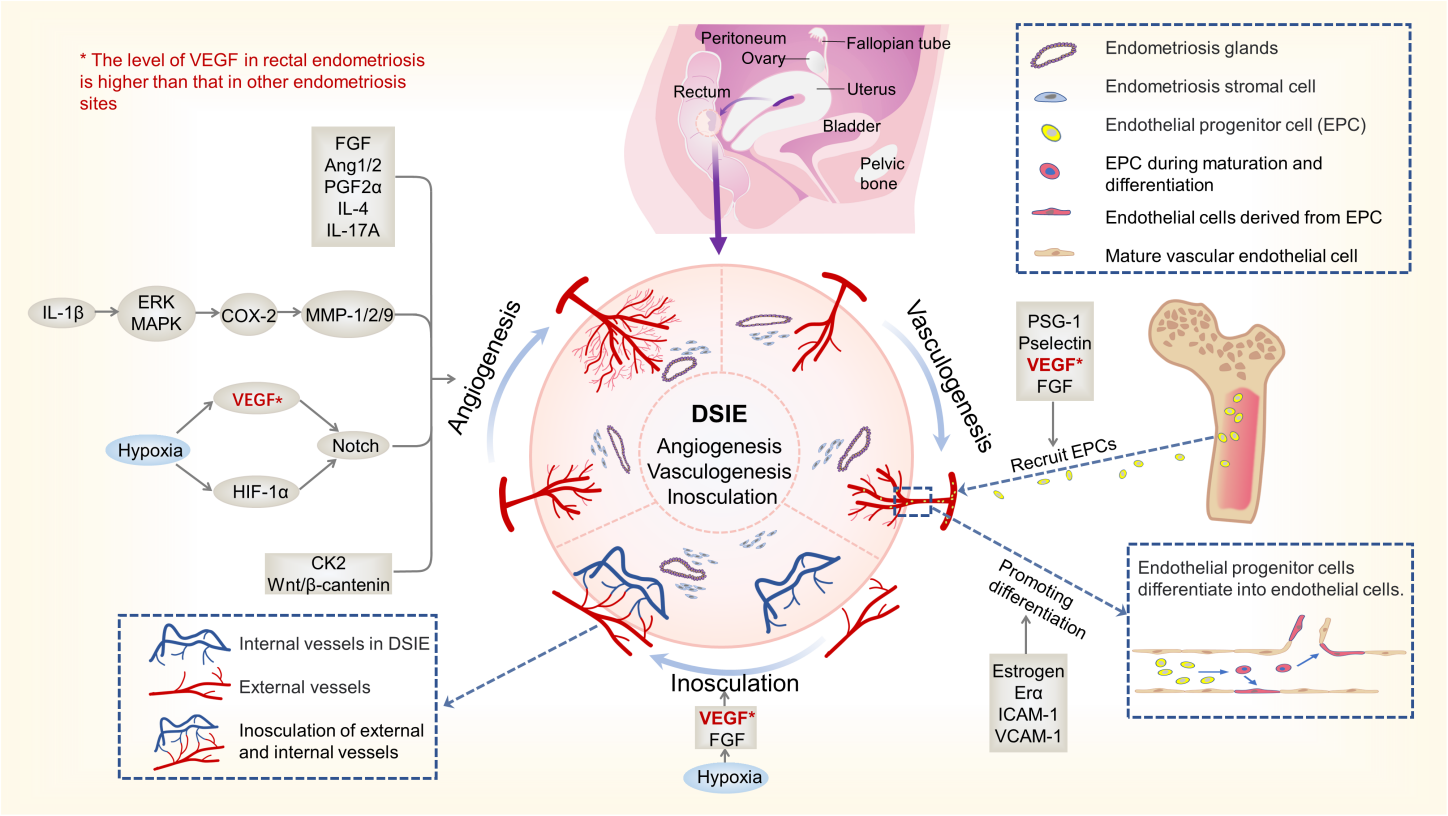
DSIE angiogenesis, including angiogenesis, vasculogenesis and inosculation. Serum VEGF levels can be upregulated in response to HIF‐1α and COX‐2 stimulation, which negatively feedback inhibits the notch pathway and simultaneously regulates angiogenesis. EPCS recruits to endometriosis lesions by VEGF, FGF and other factors, differentiates and adheres to form neovascularization by oestrogen, ICAM‐1 and VCAM‐1. Finally, VEGF stimulates the process of Inosculation, which leads to the connection between each neovascularization or between neovascularization and existing vessels. Among them, the important factor VEGF was abundantly expressed in DSIE.

Significant vascularization in and around the endometriotic tissue. Similar to endometrial tissue,^72^ the vascularization of endometriotic lesions is affected by hormonal changes in the body that vary dynamically with the menstrual cycle.^73^ This includes the generation of new blood vessels from pre‐existing vessels, called ‘angiogenesis’^74^; the differentiation and assembly of precursor cells of angiogenic cells into new vessels, called ‘vasculogenesis’,^75^ and the interconnection of blood vessels, called ‘inosculation’.^76^ This process involves interactions between hypoxia, hormones, multiple cytokines and multiple signalling pathways to regulate angiogenesis in endometriotic tissue^73^ (Figure 1). Hypoxia in diseased tissues induces upregulation of hypoxia factor hypoxia‐inducible factor‐1α (HIF‐1α), which in turn promotes increased VEGF content, and excess VEGF regulates vascular endothelial cell production together with the notch signalling pathway.^77^, ^78^, ^79^ COX‐2 can link angiogenesis in endometriotic tissue to inflammation, and COX‐2, which is regulated by the EPK/MAPK pathway, can promote increased VEGF and activation of MMP‐1,2,9, promoting Angiogenesis.^80^, ^81^, ^82^ Additionally, CK2 and catenin are involved in angiogenesis in diseased tissues.^83^ Vasculogenesis is associated with endothelial progenitor cell (EPCs) recruitment and differentiation. Increased serum levels of VEGF, fibroblast growth factor (FGF), P‐selectin glycoprotein ligand‐1 (PSGL‐1) and P‐selectin promote the migration of EPCs from the bone marrow to endometriosis lesions.^84^, ^85^, ^86^ VEGF and fibroblast growth factor (FGF) are important factors that promote Inosculation.^87^ EPCS that migrate into the lesion differentiate into vascular endothelial cells in response to oestrogen and Era, simultaneously bind to ligands intercellular adhesion molecule‐1 (ICAM‐1) and vascular cell adhesion molecule‐1 (VCAM‐1) and adhere to form neovascularization.^88^, ^89^ Endometriosis involving the rectum has been shown to increase angiogenesis in the stroma of the lesion and has a significantly higher density of VEGF and its receptor VEGFR‐2 than other types of endometriosis.^78^ Vascular endothelial growth factor (VEGF) is an important regulator of angiogenesis. Serum and peritoneal fluid is also significantly elevated in patients with DSIE. Interestingly, after surgical excision of the DSIE tissue, normalization of VEGF levels in both the serum and abdominal fluid and an increase in sVEGFR‐2, which is a negative regulator of VEGF, were observed.^90^ Hypoxia and IL‐8 are potent stimulators of VEGF upregulation.^91^, ^92^, ^93^ However, one study pointed out that the expression of HIF‐1α in digestive endometriosis did not differ from other types of lesions.^94^ In contrast, IL‐8 is more highly expressed in DIE than in other types of lesions.^95^ In summary, DSIE may have a more significant vascularization response than other types of endometrioses. However, specific molecular mechanisms are required.

### 6.3 Nerve recruitment phenomenon

**FIGURE 2:**
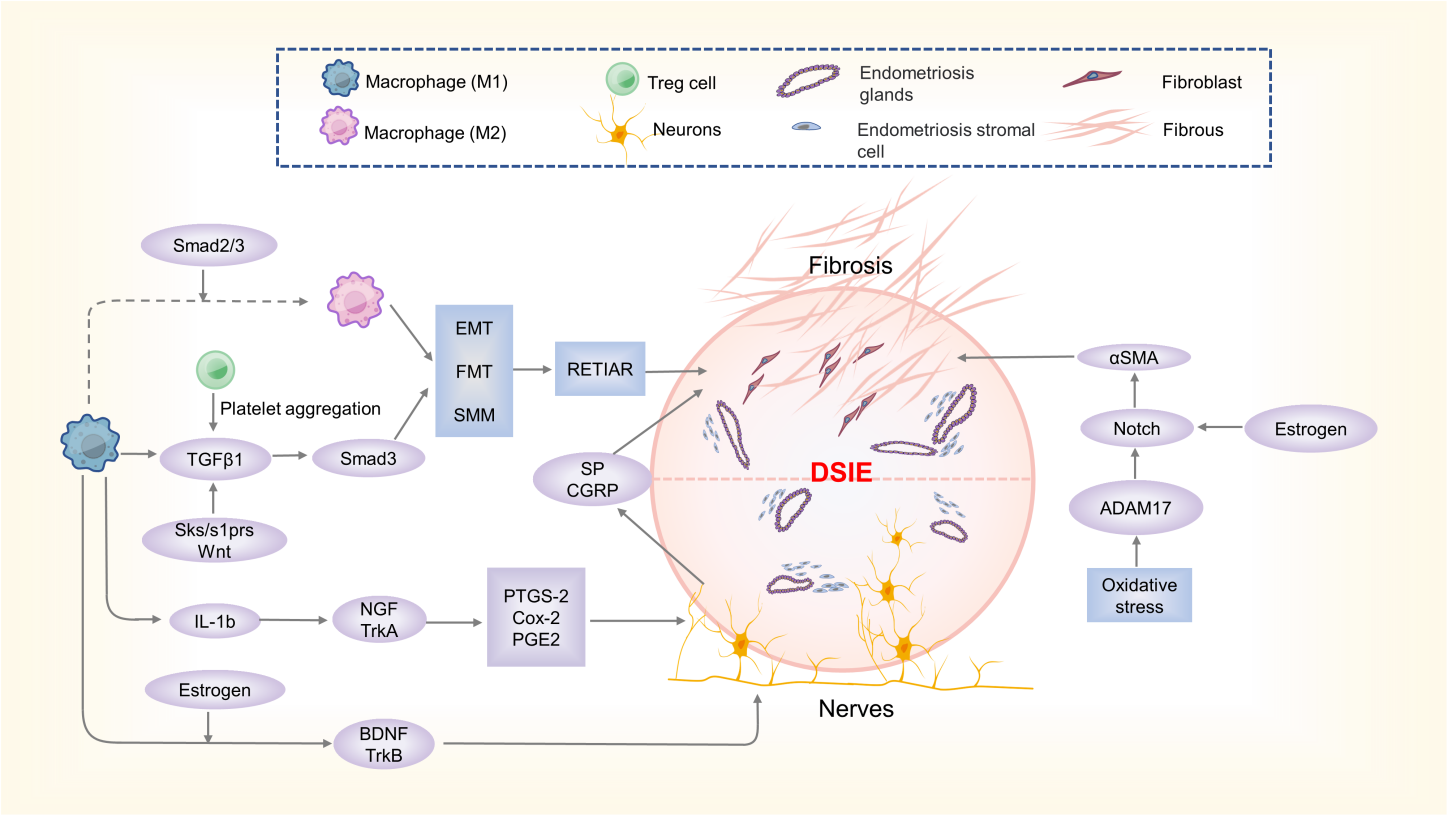
Mechanisms of nerve and fibre tissue formation and their interactions in DSIE. BDNF secreted by M1 macrophages in response to oestrogen interacts with the receptor TrkB to promote peripheral nerve growth in the lesion. NGF‐TrkA pathway, on the contrary, can promote nerve recruitment. TGF‐β1 secreted by M1 macrophages activates Smad3, and M1 macrophages are induced by the Smad2/Smad3 pathway to transform into M2 macrophages, which together with TGF‐β1 activate ReTIAR. Simultaneously, oxidative stress in the lesion activates the notch pathway and promotes lesion fibrosis. Additionally, nerve fibres enriched in the lesion can promote fibre formation via SP, CGRP.

Nerve recruitment has also been observed in DSIE lesions. Moreover, the density of nerve fibres in intestinal endometriosis is significantly higher than that in other types of lesions, and most of these nerve fibres are sensory A‐delta/C, cholinergic and adrenergic nerve fibres.^96^, ^97^ This phenomenon may explain why patients with intestinal endometriosis exhibit more severe deep pain.^98^, ^99^, ^100^ Rectal endometriosis is more likely to occur in nerve‐rich areas, suggesting that nerve recruitment may be associated with lesion invasion infiltration.^101^ Furthermore, recent studies have found that nerve fibres in intestinal endometriosis are denser around diseased nodes than around normal nodes.^102^ This reinforces the association between neural recruitment and aggressiveness of DSIE. Neural recruitment is believed to be regulated by several neurotrophic factors and neuroimmune interactions.^103^ (Figure 2). Nerve growth factor (NGF) and its receptor tropomyosin‐related kinase A (TrkA) are upregulated in DSIE lesions, especially when the lesions are neural.^104^, ^105^ Elevated IL‐1β in lesions has been shown to induce NGF expression.^106^ The NGF‐TrkA pathway affects nerve recruitment and growth by stimulating prostaglandin‐endoperoxide synthase 2 (PTGS‐2)/cyclooxygenase 2 (COX‐2) and prostaglandin E2 (PGE2).^105^ Brain‐derived neurotrophic factor (BDNF) and its receptors, tropomyosin‐related kinase B (TrkB) and P75 was found to be more highly expressed in tissues with intestinal endometriosis lesions than in other types of endometriosis.^107^ BDNF is mainly secreted by macrophages and is regulated by oestrogen in endometriosis lesions.^53^ Significantly elevated oestrogen levels can modulate neural recruitment in intestinal endometriosis in this way.^102^ In addition, synaptophysin (SYP) regulates the release of neurotransmitters, which may be related to the symptoms of deep pain in DIE patients.^108^, ^109^ Interestingly, transient receptor potential vanilloid 1 (TRPV1) is a non‐selective cation channel that is upregulated in rectosigmoid endometriosis and may be associated with difficult bowel movements and deep pain in such patients.^110^

In summary, significant nerve recruitment occurred around DSIE lesions. This neural recruitment phenomenon can be regulated by various factors such as NGF, BDNF and neuroimmune interactions. Mast cells produce an inflammatory environment that mediates peripheral neurogenesis and are closely associated with pain symptoms.^111^ Nerve recruitment is associated with infiltrative metastasis in most bowel endometriosis cases, and endometriotic lesions that metastasize to the bowel promote nerve recruitment in a variety of ways, as described above.

### 6.4 Fibrosis

DSIE differs from other types of endometriosis because its main component is fibrous rather than endometrial.^49^ These fibrous tissues form infiltrating nodules along with the endometrial tissue also in DIE.^13^ Fibrosis has long been recognized as an important part of endometriosis.^112^ Fibrosis of endometriotic tissue is thought to be linked to ReTIAR hypothesis (repeated tissue injury and repair (ReTIAR) hypothesis, which promotes epithelial‐mesenchymal transition (EMT), fibroblast‐to‐myofibroblast transdifferentiation (FMT) and smooth muscle metaplasia (SMM), resulting in fibrous formation.^113^ This mechanism is similar to that observed in cancer. Multiple cellular components are involved in ReTIAR^114^ (Figure 2). The ReTIAR mechanism is amplified by the cyclic alterations of diseased tissues and the surrounding microenvironment, where oxidative stress is exacerbated. TGF‐β1 is an essential player in the formation of fibrosis in and around endometriotic tissue.^114^, ^115^, ^116^ The increase in TGF‐β1 expression in DSIE tissues has been described above. First, platelet aggregation is promoted by Tregs in the lesion tissue, and aggregated platelets can activate the TGF‐β1/Smad3 pathway, promoting ReTIAR in the lesion tissue and leading to fibrous tissue production.^117^, ^118^ The Smad2/Smad3 pathway induces macrophage polarization from M1 to M2.^119^ M1 macrophages have pro‐inflammatory effects. M2 macrophages promote tissue repair and fibrosis.^120^ Macrophages can also secrete important factors such as TGF‐β1, IL‐6, VEGF, etc.^121^ TGF‐β1 acts on the Smad3 pathway. TGF‐β1 can also be produced by mesenchymal stem cells (MSCs) via the Wnt/β‐linked protein pathway paracrine secretion.^122^ Recent studies have shown that elevated IL‐6 levels result in sustained engagement of the signal transducer and activator of the transcription 3 (STAT3) pathway, thereby promoting fibrosis in ectopic endometrial tissue.^123^ IL‐10 acts as an anti‐inflammatory and antifibrotic agent via the STAT3 pathway in DED‐related organs. However, IL10 promotes fibrosis through STAT‐3 as well as IL‐6, which must be considered in the treatment of DIE.^124^ Recently, sensory nerve fibres were found to promote fibrosis of endometriotic lesions in mice.^125^ This may be because the neuropeptide substance P (SP) and calcitonin gene‐related peptide (CGRP) in sensory nerve fibres pass through NK1R/CGRP/CRLR / and RAMP‐1 signalling promotes ReTIAR.^126^ This partly explains the presence of more neural and fibrous tissues in DSIE lesions. The sphingosine kinase (SKs) and specific S1P receptors (S1PRs) pathways also cause fibrosis by promoting TGFβ1.^127^ Moreover, the specific S1P transporter Spns2, which is essential for the release of S1P into the extracellular space, was only upregulated at DIE.^127^ Compared with other types of endometriosis, more endometrial stromal cells participate in the fibrosis of DIE lesions.^112^ Oxidative stress also contributes to the increased expression of ADAM17 and AOPPs, which can bind to the Notch complex, which can also be activated by elevated IL‐6 in the lesion via E‐proteins on the cell membrane of lesions and activate the transcription of fibrosis‐related genes.^43^, ^128^, ^129^ Moreover, the expression of the AKT and ERK signalling pathways significantly increases in fibrotic DIE lesions,^130^ which may be related to the fact that the growth of DIE tissue is not inhibited by the surrounding fibrous tissue.^130^ Additionally, periostin and its upstream factor transcription factor 21 (TCF21)^131^ as well as the Wnt/β‐Catenin pathway activated by Forkhead box protein P1 (FOXP1)^132^ also play an unclear role in fibrosis.

### 6.5 Aggressiveness and malignant transformation of DSIE

Endometriosis has a malignant tumour‐like aspect, especially the aggressiveness exhibited by DSIE.^133^, ^134^ Intestinal endometriosis is often multifocal and multicentric.^135^ A tumour‐like appearance can sometimes be misdiagnosed as a malignant tumour of the digestive tract.^136^ The malignant aggressiveness of DSIE is associated with collective cell migration (CCM) and EMT.^137^, ^138^ CCM regulates tissue repair and renewal and participates in cancer spread.^139^ Previous studies have suggested that during invasion, polarized epithelial cells lose intercellular adhesion and transform into single, highly motile mesenchymal cells, contributing to the invasive process of DSIE. This process is regulated by a significant increase in the EMT‐induced transcription factor ZEB‐1 in epithelial cells of diseased tissues.^138^ N‐Cadherin, a mesenchymal marker, activates the MAPK–ERK pathway, which leads to increased transcription of MMP9 and promotes the invasion of lesions.^140^ Olivier found that in the intestinal proliferative nodules of DSIE, the outwardly invasive part of the glandular tissue became thinner, cell mitosis was more active, the level of E‐cadherin expression decreased but was not lost, and the level of N‐cadherin expression was significantly enhanced. This indicates that more than EMT may be involved in the invasion of DSIE because cell–cell adhesion does not completely disappear, which indicates that CCM may be significantly involved.^137^, ^141^ It is worth mentioning that the opposite result was found in Van's study, in which N‐cadherin expression was significantly reduced in tissues with more aggressive gastrointestinal endometriosis lesions.^142^ This may be because ectopic endometrial tissue undergoes cyclical changes similar to normal endometrial tissue, implying that the aggressiveness of DSIE varies with the endometrial cycle. In addition, the density of nerve fibres was higher in the glands before the invasion of the lesion tissue, suggesting that aggregated nerve fibres may also affect the invasiveness of the lesion.^143^ In addition, other mechanisms may be related to the increased invasiveness of DSIE, such as a positive correlation between the expression of Kisspeptins and CA‐125.^144^

Mutations in somatic cancer drivers are found in most DSIE lesions. However, as the relationship between such mutations and the associated cancers remains unclear, Guo cautiously referred to them as cancer‐associated mutations (CAMs).^145^ Ectopic endometrial tissue, such as orthotopic endometrial tissue, undergoes cycles of tissue damage repair as well as remission of inflammation and oxidative stress.^146^ This leads to the appearance of CAMs in the endometriotic tissue cells.^147^ Interestingly, the degree of genetic alteration in the diseased tissue may be related to the distance of the diseased tissue from the normal endometrium,^148^ suggesting that the diseased tissue may have originated from the normal tissue by regulating CAMs. During ectopic development, orthotopic cells in the presence of CAMs have a selective advantage and participate in the ectopic process; however, the details are still lacking.^149^ CAMs in diseased tissues usually contain AT‐rich interactive domain‐containing protein 1A (ARID1A), Phosphatidylinositol‐4,5‐Bisphosphate 3‐Kinase Catalytic Subunit Alpha (PIK3CA), Protein Phosphatase 2 Scaffold Subunit A alpha (PPP2R1A) and Kirsten rat sarcoma viral oncogene (KRAS).^150^ Among these, the activation of KARS is present only in DIE,^151^ which encodes a small GTPase essential for cell proliferation.^152^ These CAMs also promote fibrosis in lesioned tissue.^113^, ^145^ Additionally, PIK3CA, ARID1A and KARS are associated with the malignant transformation of lesions.^153^, ^154^ However, the probability of malignant transformation due to CAMs is extremely low, and only a small number of untreated endometriosis cases turn into malignancy.^145^, ^155^

## 7 TREATMENT OF DSIE

The clinical presentation of DSIE is often atypical, making its diagnosis challenging.^156^ According to the European Society for Human Reproduction and Embryology (ESHRE) 2022 guidelines, hormone therapy (combined hormonal contraceptives, progestogens, gonadotropin‐releasing hormone agonists or gonadotropin‐releasing hormone antagonists) and surgery can both effectively reduce pain symptoms in patients with pelvic endometriosis. No prognostic markers are available to select patients who would benefit from surgery, indicating that surgery is not always the best treatment option. Clinicians should adopt a shared decision‐making approach to individualize the treatment plan by comprehensively evaluating side effects, personal preferences, cost, efficacy and the need for pregnancy. However, for abdominal extrapelvic endometriosis, the guidelines recommend that surgery should be preferred to ease the symptoms of patients as much as possible.^156^, ^157^, ^158^, ^159^ However, surgical treatment has some limitations. To address symptoms such as gastrointestinal bleeding and deep pain caused by DSIE, surgery often involves removing parts of the intestine, bile ducts and other organs, which often cause damage to the patient. Recently, the use of indocyanine green and fluoroscopic laparoscopy has been reported to identify diseased nodules and reduce surgical harm.^160^ Conventional oestrogen and progestin treatments often only alleviate symptoms such as deep pain and have limited effects on the root cause of the lesion.^161^, ^162^ GnRHa is effective in reducing the inflammatory response and production of blood and lymphatic vessels in endometriosis but is not effective in DSIE.^163^ Researchers have attempted to address this dilemma by using emerging drugs to address the problem of multiple surgeries in patients. Prostaglandin E2 (PGE) and the receptors, EP2 and EP4, can act inversely on fibrosis in endometriosis, while α7 nicotinic acetylcholine receptor (α7nAChR) and tanshinone IIA sulfonate can also inhibit fibrosis, which may provide a new therapeutic target.^164^, ^165^, ^166^, ^167^ A number of targeted drugs have been found to be beneficial in the treatment of endometriosis, including tesilomox, which inhibits oxidative stress in the lesion by inhibiting the ERK and mTOR/AKT pathways^44^; Leflunomide, which inhibits NF‐κB; Sorafenib, which inhibits cell proliferation activity, and α‐NETA, which inhibits the chemokine/CMKLR1 signalling axis.^168^, ^169^, ^170^ Thus, these drugs can be used as novel drugs for the treatment of endometriosis. However, most of these drugs are currently in the animal testing stage, and their effects and side effects in humans remain to be studied.

## 8 FUTURE PERSPECTIVES

In conclusion, DSIE is an uncommon form of endometriosis warranting further investigation. DSIE has a unique clinical presentation, biological behaviour and pathophysiological mechanisms that are different from those of general endometriosis, making the diagnosis and treatment of DSIE clinically difficult. Surgery remains the best treatment for these conditions. The current understanding of DSIE remains limited. Researchers tend to study endometriosis only in the intestine, particularly in the rectosigmoid colon. Endometriosis in the liver, biliary tract and other organs of the digestive system has not been effectively studied. A major problem is that the organ model is not repeatable for other tissue locations, and the molecular mechanisms involved may be completely different. However, it is not known whether they have a different physiological mechanism from intestinal endometriosis. Further detailed studies on endometriosis in other organs of the digestive system should be conducted. Furthermore, current research has identified a variety of drugs that can reduce inflammation and fibrosis in DSIE to ease pain but often have limited effects on the radical treatment of the lesion. Emerging drugs are still in the animal testing stage and lack clinical application. Although immunotherapy is important, a specific targeting method must be established. Future research on nonsurgical methods of radical treatment of DSIE is required to cure this painful disease.

## AUTHOR CONTRIBUTIONS

**Wenze Yin:** Data curation (equal); writing – original draft (equal). **Xiaoqing Li:** Data curation (equal); visualization (lead); writing – original draft (equal). **Peng Liu:** Resources (supporting); writing – review and editing (equal). **Yingjie Li:** Resources (supporting); writing – review and editing (supporting). **Jin Liu:** Resources (supporting). **Shan Yu:** Conceptualization (equal); funding acquisition (equal); writing – review and editing (equal). **Sheng Tai:** Conceptualization (equal); project administration (equal); writing – review and editing (equal).

## FUNDING INFORMATION

This study was supported by the Research Project of the China Primary Health Care Foundation.

## CONFLICT OF INTEREST STATEMENT

The authors declare no conflicts of interest.
